# Intimal and medial arterial changes defined by ultra-high-frequency ultrasound: Response to changing risk factors in children with chronic kidney disease

**DOI:** 10.1371/journal.pone.0198547

**Published:** 2018-06-14

**Authors:** Frida Dangardt, Marietta Charakida, Scott Chiesa, Devina Bhowruth, Alicja Rapala, Daniela Thurn, Franz Schaefer, John Deanfield, Rukshana Shroff

**Affiliations:** 1 Vascular Physiology Unit, National Centre for Cardiovascular Prevention and Outcomes, Institute of Cardiovascular Science, University College London, London, United Kingdom; 2 Department of Pediatric Clinical Physiology, The Queen Silvia Children’s Hospital, The Sahlgrenska Academy and University Hospital, Gothenburg, Sweden; 3 Division of Imaging Sciences & Biomedical Engineering, King's College London, London, United Kingdom; 4 Department of Pediatric Nephrology, Hannover Medical School, Hannover, Germany; 5 Division of Pediatric Nephrology, Center for Pediatrics and Adolescent Medicine, University of Heidelberg, Heidelberg, Germany; 6 Nephrology Unit, Great Ormond Street Hospital for Children NHS Foundation Trust, London, United Kingdom; Michigan State University, UNITED STATES

## Abstract

**Background:**

Patients with chronic kidney disease (CKD) are exposed to both traditional ‘Framingham’ and uremia related cardiovascular risk factors that drive atherosclerotic and arteriosclerotic disease, but these cannot be differentiated using conventional ultrasound. We used ultra-high-frequency ultrasound (UHFUS) to differentiate medial thickness (MT) from intimal thickness (IT) in CKD patients, identify their determinants and monitor their progression.

**Methods:**

Fifty-four children and adolescents with CKD and 12 healthy controls underwent UHFUS measurements using 55-70MHz transducers in common carotid and dorsal pedal arteries. Annual follow-up imaging was performed in 31 patients.

**Results:**

CKD patients had higher carotid MT and dorsal pedal IT and MT compared to controls. The carotid MT in CKD correlated with serum phosphate (p<0.001, r = 0.42), PTH (p = 0.03, r = 0.36) and mean arterial pressure (p = 0.03, r = 0.34). Following multivariable analysis, being on dialysis, serum phosphate levels and mean arterial pressure remained the only independent predictors of carotid MT (R^2^ 64%).

Transplanted children had lower carotid and dorsal pedal MT compared to CKD and dialysis patients (p = 0.02 and p = 0.01 respectively). At 1-year follow-up, transplanted children had a decrease in carotid MT (p = 0.01), but an increase in dorsal pedal IT (p = 0.04) that independently correlated with annualized change in BMI.

**Conclusions:**

Using UHFUS, we have shown that CKD is associated with exclusively medial arterial changes that attenuate when the uremic milieu is ameliorated after transplantation. In contrast, after transplantation intimal disease develops as hypertension and obesity become prevalent, representing rapid vascular remodeling in response to a changing cardiovascular risk factor profile.

## Introduction

Cardiovascular disease begins early in the course of chronic kidney disease (CKD)[1,2] and is a life-limiting problem, even in children [3]. Carotid artery intima media thickness (IMT) increases with uremic[1,4–7] as well as traditional Framingham risk factors[8,9], and may reflect medial changes (arteriosclerosis), intimal changes (atherosclerosis), or both. The determinants of these early vascular changes, as well as their progression and response to therapy are likely to be different[10]. While intimal disease is largely attributed to traditional cardiovascular risk factors[1,8], medial disease is caused by CKD-specific risk factors, predominantly mineral dysregulation, and manifests as vascular calcification[1,2,6].

Conventional IMT is unable to differentiate between changes in arterial intima and media. Recently, ultra-high frequency ultrasound (UHFUS) imaging has enabled visualization of the intimal and medial layers of the vessel wall in exquisite detail[11] so that medial (MT) and intimal thickness (IT) can be assessed in central and peripheral arteries[12,13] and arterial disease progression can be monitored[14,15]. One study, in adult hemodialysis patients, has reported increased radial artery IT and MT compared to healthy controls[16], but risk factors and progression have not been studied. Arterial wall changes are complex, with medial disease likely to be due to CKD-specific factors[1,17], especially mineral dysregulation, whereas intimal disease may reflect traditional cardiovascular risk factors. Vascular disease develops from the first decade of life in children on dialysis[6], presenting an opportunity for prevention. We used UHFUS to study the vascular phenotype in children and examine the impact of uremic risk factors and dialysis as well as changes in cardiovascular risk factor profile after renal transplantation.

## Patients and methods

### Study design

We recruited 54 consecutive children and adolescents with CKD stage 4–5, on dialysis or with functioning renal transplants from clinics at Great Ormond Street Hospital for Children, London. We excluded children <5 years of age (since vascular imaging can be technically challenging and no reference data are available [18]) and those with infections in the preceding 3 months before scanning. 19 children had pre-dialysis CKD stages 4–5, 20 were on dialysis and 15 children had a functioning renal transplant. Since the modality of renal replacement therapy (CKD, dialysis or transplantation) is known to strongly influence cardiovascular changes, we also performed annual follow-up in all the children who continued in the same group for 12 months: this included 31 children, 16 in pre-dialysis CKD and 15 post-transplant. Sixteen of 20 children on dialysis received a renal transplant within 1-year, and due to different intervals between transplantation and annual vascular measures, there was too much variability in this group to undertake further analysis. Demographic details, body mass index (BMI), blood pressure (BP), ambulatory blood pressure monitoring (ABPM) and CKD-specific biochemical profile are described in Table 1. The patient cohort was compared with 12 healthy age-, gender- and ethnicity- matched children. Written informed consent was obtained from all parents or caregivers and from children when appropriate. The study was approved by the local research ethics committee and conducted along the principles of the Declaration of Helsinki.

**Table 1:** Independent predictors of common carotid artery medial thickness on multivariate analysis.

Variable	Standardized coefficient (β)	p	Model R^2^
Dialysis vs CKD or transplant	0.76	<0.0001	64%
Serum phosphate level	0.48	0.02	
Mean arterial pressure standard deviation score	0.39	0.04	

### Vascular imaging

All patients had vascular imaging by both UHFUS and conventional ultrasound in the carotid and dorsal pedal arteries at the same site at baseline and follow-up.

### Ultra high-frequency ultrasound

**Fig 1:**
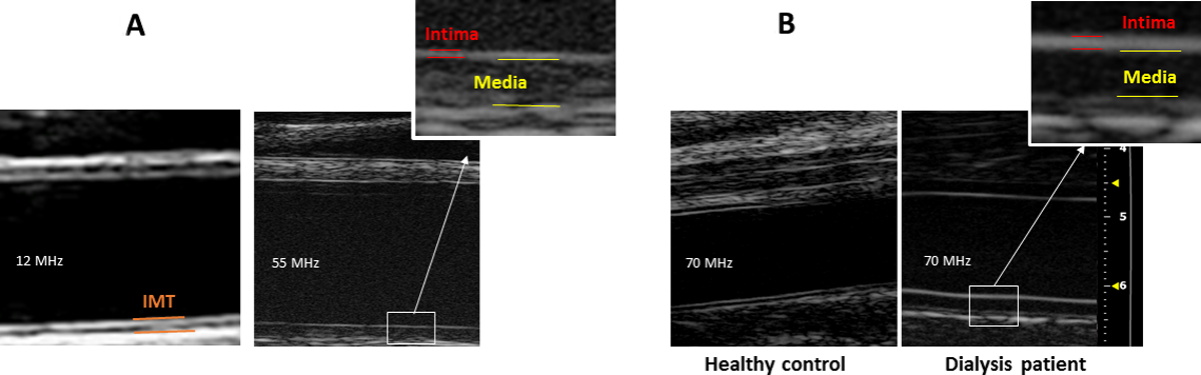
Ultra-high-frequency ultrasound images of carotid and dorsal pedal arteries, and comparison with images obtained by conventional ultrasound. **Fig 1A**—Carotid artery scanned with 12 MHz (left) and 55 MHz (right) ultrasound. Outtake shows magnification of intima-media complex from 55 MHz image with outline of intima and media thickness. **Fig 1B**—Dorsal pedal artery of control child (left) and child on hemodialysis (right) scanned with 70 MHz probe).

IT and MT were measured using UHFUS (Vevo® 2100, Fujifilm VisualSonics, Canada) with 55 and 70 MHz peak frequency linear array transducers for carotid and dorsal pedal imaging respectively (MS550 and MS700, Fujifilm VisualSonics, mean beam frequency range 22–55 MHz and 30–70 MHz respectively; Fig 1A and 1B). End-diastolic longitudinal images were acquired for at least three consecutive beats, with the IT and MT in both near and far wall clearly visible, and measured manually using calipers (Fig 1A and 1B), as no automated software existed for measuring IT and MT separately. IT was defined as the distance between the lumen-intima interface and the intima-media interface of the far wall. MT was defined as the distance between the intima-media interface and the media-adventitia interface of the far wall, as previously described ([11]; Fig 1A and 1B). All measures were performed by two observers (FD and DB) who were blinded to the clinical condition of the child. Inter-observer variability was assessed in a separate cohort of 10 adolescents (mean coefficient of variation 3.8 ± 1.0%). Intraclass Correlation Coefficient (ICC) was 0.92 and 0.76 for carotid IT and MT, respectively, and 0.82 and 0.96 for dorsal pedal IT and MT, respectively.

UHFUS imaging was performed using the highest frequency transducer that allowed clear visualization of the vessel as described above, including far wall. For carotid measurements that require a greater depth of penetration, only 6 of 54 (11%) patients could be scanned with the 70 MHz transducer, whereas 96% of carotid measurement were possible with the 55 MHz transducer. All the dorsal pedal vessels, which are more superficial, were clearly visualized with the 70 MHz transducer, and measured above the proximal part of the first metatarsal bone in the foot.

### Conventional ultrasound for carotid artery IMT

Conventional IMT measurements were performed in all children as part of the Cardiovascular Comorbidity in Childhood CKD (4C) study, a longitudinal cohort study that includes 700 children across Europe with annual cardiovascular assessments[19]. The intima-media thickness of the common carotid artery was measured by standard B-mode ultrasound using a 12 MHz (mean beam frequency range 5.6–14 MHz) linear array transducer (M12L, Vivid7, GE Medical, Horton, Norway)[6]. Longitudinal two-dimensional images of the vessel 1–2 cm proximal to the carotid bulb were acquired on the R wave of the electrocardiogram, frozen in diastole, and analyzed off-line using an automated edge detection system. The cIMT was calculated as the distance between the leading edges of the lumen-intima interface and the media-adventitia interface on the far wall of the artery. The measurement was repeated for three different cardiac cycles and the average of the three measurements was used. cIMT changes with growth, so cIMT standard deviation score (SDS) were derived using reference values normalized for height and age in European children[18]. Well-established nomograms are available for conventional cIMT[18] in children, so these measures were not performed in the controls.

### Ambulatory blood pressure monitoring

ABPM was performed in all patients at baseline and 12 months using Spacelabs 90207-2Q oscillometric devices (*Spacelabs Healthcare*) and time–averaged 24-hour mean arterial pressure (MAP) used for further comparisons. All BP readings were normalized to standard deviation scores (SDS) using European reference data[19].

### Biochemistry

Blood tests and vascular measures were performed at the same clinic visit: before a mid-week session of hemodialysis or at clinic review for predialysis CKD, peritoneal dialysis, and transplant recipients. Routine non-fasting blood samples were collected and serum was frozen at -80°C; all analyses were performed in a blinded fashion. We did not have ethical approval to perform blood tests in the healthy controls. The following biochemical analyses were performed in all patients at baseline and 12-month follow-up: renal function tests, measures of mineral dysregulation (calcium, phosphate, parathyroid hormone [Immulite 2500 Intact PTH assay; *Siemens Healthcare Diagnostics*]), 25(OH)D (isotope-dilution liquid chromatography-tandem mass spectrometry), high-sensitivity CRP (Human SimpleStep ELISA, *Abcam*, UK), lipid profile (total cholesterol, HDL and LDL cholesterol, and triglycerides [colorimetric enzymatic method; *Technicon* automatic analyzer RA-1000; Dade Behring, Germany]), FGF23 (second generation human FGF23 C-Terminal ELISA, *Immutopics International*, San Clemente, CA), s-klotho (solid-phase sandwich ELISA, *Immuno-Biological Laboratories Co*. *Ltd*, Gunma, Japan). Estimated GFR was calculated using the modified Schwartz formula. Serum calcium, phosphate and PTH levels are described as mean time-averaged levels over one year.

### Statistical analyses

Data are presented as mean ± SD or median (range) as appropriate. Univariate comparisons of continuous variables between the groups (CKD vs dialysis vs transplant) were performed by one-way ANOVA. Comparisons of continuous variables between baseline and final follow-up were performed using a paired *t-*test or the non-parametric Wilcoxon test as appropriate. For multiple comparisons of several groups, the Kruskal–Wallis test was performed. Correlation analyses were undertaken using Spearman’s test. The level of agreement between carotid IMT by conventional ultrasound and by UHFUS was calculated by Bland-Altman analysis. Two multivariable regression analyses models were built for factors independently influencing carotid MT and dorsal pedal IT, and all variables known to influence vascular measures were included, provided they were significant on univariate analyses with p value <0.15. Variables which were not normally distributed, including s-klotho, 25(OH)D and PTH were log-transformed to achieve normality. All analyses were performed with GraphPad Prism (version 7.0; *GraphPad Software Inc*) or SPSS (version 23, IBM SPSS Statistics, IBM Corp), and a two-tailed p<0.05 was considered statistically significant.

## Results

### UHFUS imaging in different vascular beds

**Fig 2:**
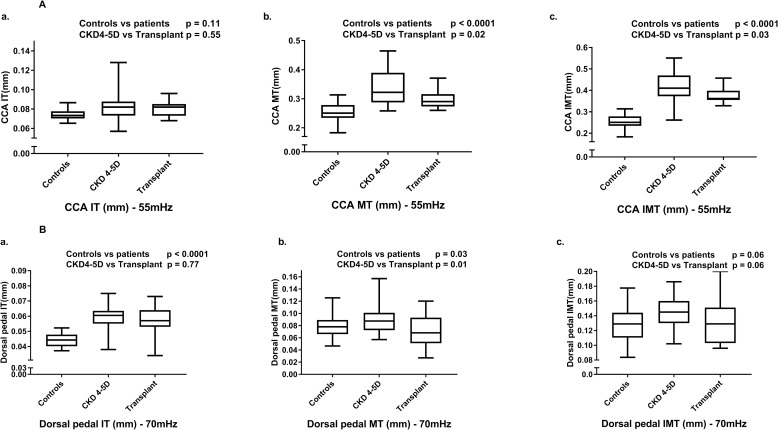
Ultra-high-frequency ultrasound measurements of intima thickness (IT), medial thickness (MT) and the composite measure of IMT in carotid (Fig 2A) and dorsal pedal (Fig 2B) arteries in healthy controls and children with CKD4-5 and on dialysis (CKD4-5D) and transplanted patients. CCA–common carotid artery.

Carotid MT was higher in patients compared with controls (p<0.0001; Fig 2A), but there was no difference in carotid IT. In the dorsal pedal artery, both MT and IT were higher in the patient group (p = 0.03 and p<0.0001 respectively; Fig 2A and 2B). Within patient groups, the CKD and dialysis patients had an increased MT in carotid (p = 0.02) and dorsal pedal (p = 0.01) arteries compared to transplanted patients (Fig 2A-b and 2B-b). No differences in IT were noted between patient groups in any of the vascular beds.

### Determinants of increased medial thickness (MT)

The clinical characteristics of the study population is provided in S1 Table. Patients had lower BMI SDS (p = 0.03) and higher systolic and diastolic BP SDS (p = 0.02 for both) compared with controls. In the combined CKD and dialysis cohort, serum phosphate (p = 0.02), PTH (p = 0.01) and FGF-23 (p <0.001) were higher whereas soluble klotho levels were lower (p = 0.04) compared to transplanted patients. Transplanted patients had a higher BMI SDS (p = 0.003) and also a higher 24-hour mean arterial pressure (MAP) SDS compared to the CKD and dialysis cohorts (p = 0.01).

### Common carotid artery MT

**Fig 3:**
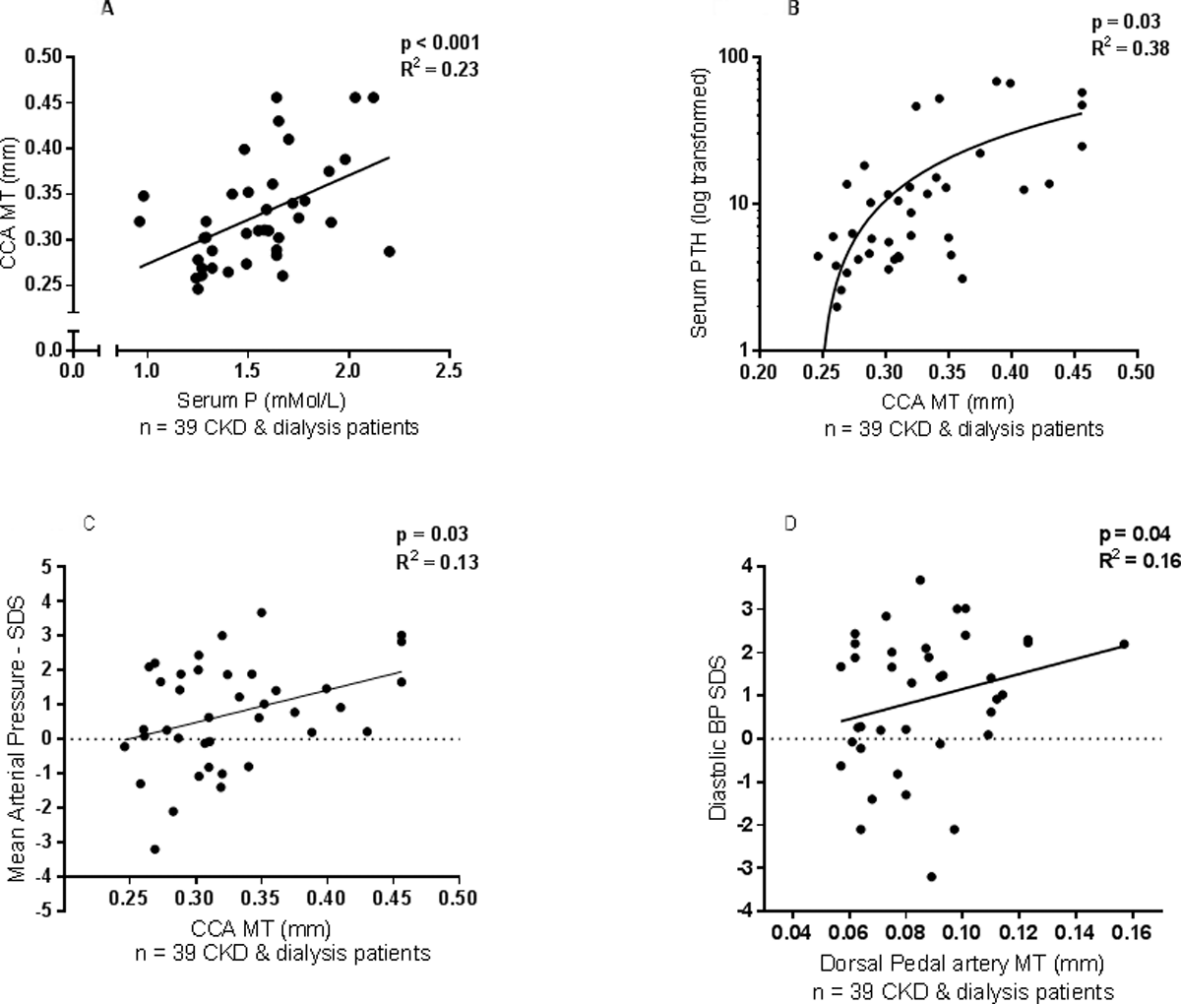
Correlation of ultra-high-frequency ultrasound measures with biochemical markers. **(Fig 3A–**Association of serum phosphate with common carotid artery (CCA) medial thickness (MT), **Fig 3B–**Association of serum parathyroid hormone level (log transformed) with CCA MT, **Fig 3C–**Association of mean arterial pressure standard deviation score (SDS) with CCA MT, **Fig 3D–**Association of diastolic BP SDS with dorsal pedal artery MT).

In the combined CKD and dialysis cohort (n = 39), the mean carotid MT correlated with time-averaged serum phosphate (p<0.001, R^2^ = 0.23) and PTH levels (p = 0.03, R^2^ = 0.38) over the preceding year and the MAP SDS (p = 0.03, R^2^ = 0.13; Figs 3A–3C). Increased carotid MT was found in children on dialysis for >1 year compared to those on dialysis for <1 year (p = 0.007).

On multivariable regression analysis, being on dialysis (as compared to pre-dialysis CKD or transplant), serum phosphate and MAP SDS were significant and independent predictors of a higher carotid MT, explaining 64% of the variance in carotid MT (Table 1).

### Dorsal pedal artery MT

The dorsal pedal artery MT in CKD and dialysis patients weakly correlated with diastolic BP SDS (p = 0.04, R^2^ = 0.16; Fig 3D), but not with any biochemical measures. In transplanted patients at baseline, there were no correlations between vascular measures and any clinical or biochemical measures.

None of the vascular measures in either controls or CKD patients at baseline correlated with age, gender, BMI SDS, serum calcium, 25(OH)D, FGF23 or soluble-klotho levels, serum cholesterol or triglycerides, or treatment with phosphate binders or vitamin D analogues.

### Risk factor profile and vascular changes at annual follow up

A 1-year follow-up, vascular measurements were examined in two groups: (i) patients who remained in pre-dialysis CKD (n = 16) and (ii) patients who were transplanted (n = 15); S2 Table.

### Risk factor profile

Over 12 months, CKD patients had an increase in serum phosphate, FGF23 and MAP SDS (p = 0.02, p = 0.003 and p = 0.043 respectively). In the transplanted patients, over the same time period of 12 months, there was an increase in BMI SDS, systolic BP SDS and MAP SDS and serum cholesterol level (p = 0.015, p = 0.04, p <0.001 and p = 0.03 respectively), but no change in uremic risk factors such as serum phosphate or PTH.

### Vascular changes

**Fig 4:**
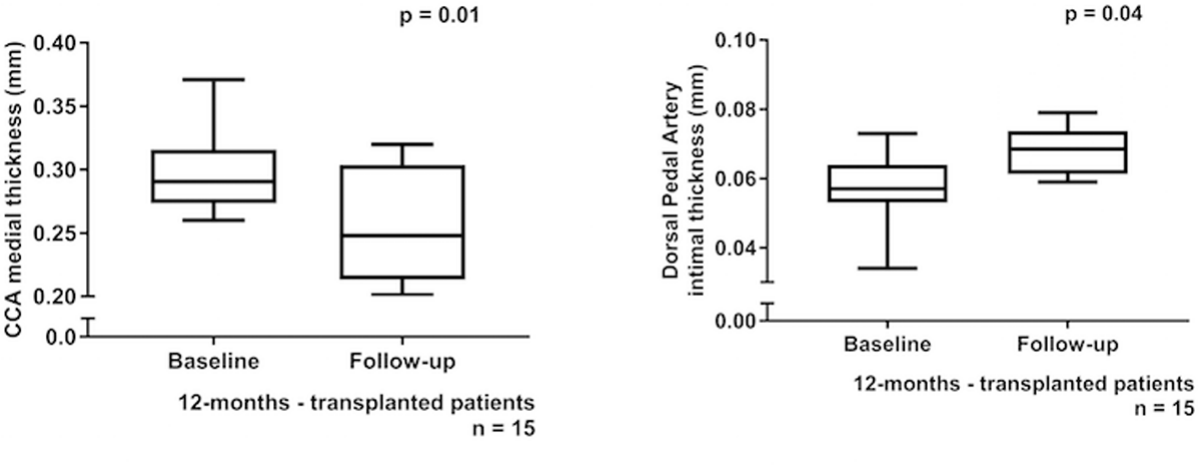
Changes in ultra-high-frequency ultrasound measures at one year follow-up. **(Fig 4A**—Comparison of baseline and 1-year follow-up measures of common carotid artery (CCA) medial thickness (MT) in transplanted children, **Fig 4B**—Comparison of baseline and 1-year follow-up measures of dorsal pedal artery intimal thickness (IT) in transplanted children).

Children and adolescents who remained in pre-dialysis CKD did not show changes in any vascular measures at annual follow-up. In contrast, transplanted patients had a decrease in carotid MT (p = 0.01; Fig 4A and S2 Table) which correlated positively with the annualized change in MAP SDS (p = 0.03, r = -0.43). Also, transplanted patients had an increase in carotid and dorsal pedal IT (p = 0.06 and p = 0.04 respectively; Fig 4B and S2 Table), which was positively associated with systolic BP SDS at follow-up (p = 0.02, r = 0.22), annualized change in MAP SDS (p = 0.05, r = 0.12) and the annualized change in BMI SDS (p = 0.4, r = 0.37). On stepwise linear regression analysis, the change in dorsal pedal IT showed a weak but independent correlation with the annualized change in BMI SDS (p = 0.04, β = 0.11, model R^2^ 29%). No change in conventional cIMT was seen on annual follow-up in either CKD or transplant groups.

### UHFUS measurement and correlation with conventional ultrasound

**Fig 5:**
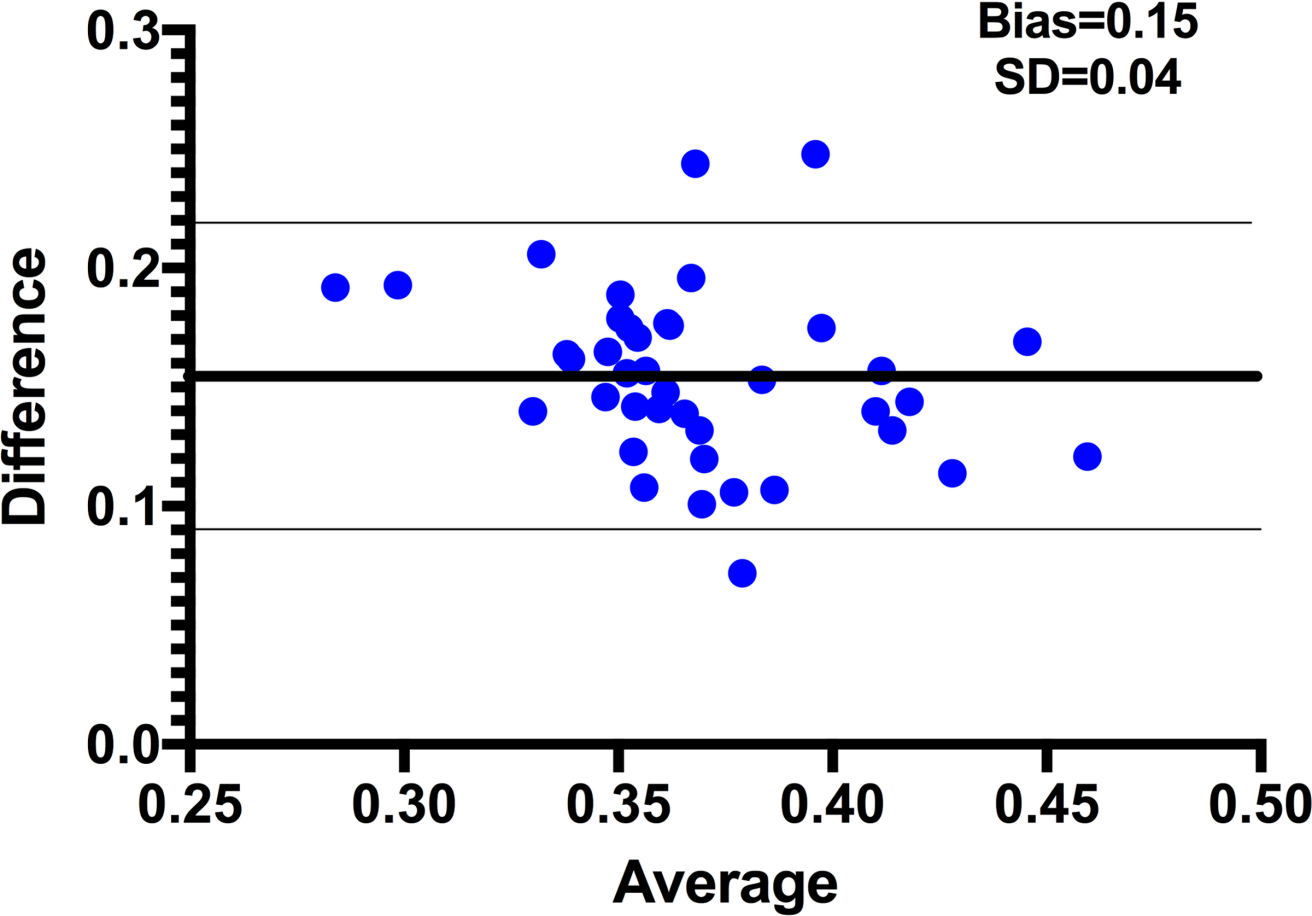
Bland Altman plot comparing the composite measure of intima thickness and medial thickness (IT + MT) by ultra-high-frequency ultrasound with conventional cIMT.

UHFUS images were compared with conventional carotid IMT measurements in all patients. Using conventional ultrasound, the carotid IMT was significantly higher in dialysis compared to transplanted patients (p<0.001). Carotid IMT by conventional ultrasound showed an overestimation of IMT by 0.15 ± 0.04 mm for conventional ultrasound compared to the sum of carotid IT and MT on UHFUS (95% confidence interval = 0.08–0.22; Fig 5. CIMT by conventional ultrasound showed a positive association with time on dialysis (p = 0.04, r = 0.23) but did not correlate with BP or any biochemical measures.

## Discussion

In this study, using UHFUS, we describe for the first time medial and intimal arterial wall changes that occur in central and peripheral arteries in children with CKD, and the relation with their complex risk factor profiles. Vascular changes in children with CKD and on dialysis were confined to the media and related to uremic risk factors such as high phosphate and PTH levels, as well as blood pressure, and increased with time on dialysis. After renal transplantation, when the uremic milieu was ameliorated, medial changes improved, but hypertension and obesity became prevalent, and intimal disease developed. These findings provide support for the use of UHFUS to prioritize treatment of specific risk factors and, with serial imaging, to determine response to treatment.

Arterial disease develops early in the course of CKD, and progresses rapidly on dialysis. Studies have shown that 20% of children on dialysis develop vascular calcification[4–6], which can begin as early as the first decade of life[6]. This contributes to an extremely high mortality risk; 40% of deaths in young adult recipients of dialysis are due to cardiovascular or cerebrovascular causes[5]. Two distinct types of arterial disease have been demonstrated in CKD: atherosclerosis affecting the intima and arteriosclerosis affecting the media, predominantly with calcification[1,2]. Intimal and medial disease may coexist in adults on dialysis [10], and are likely to have different risk factor profiles, clinical consequences and outcomes. Intimal calcification is associated with ischemic heart disease, while medial calcification increases vascular stiffness, systolic hypertension, and left ventricular hypertrophy[6,20,21]. When patients in CKD or on dialysis are transplanted their risk profile changes, with amelioration of uremia related risk factors but a greater prevalence of obesity, dyslipidemia and hypertension, as seen in our and other studies[1,22,23]. We now show that this can lead to vascular remodeling within a year, and this may explain the very high mortality from cardiovascular disease that persists even after transplantation. In children and young adults, 22% of deaths in transplant recipients are due to cardiovascular disease[3].

UHFUS is a novel high frequency ultrasound technique that was originally developed for experimental studies in small animals[24], and has recently been used in humans to detect subclinical vascular disease. With a discrimination power of 20 µm, UHFUS allows visualization of separate layers of the vessel wall (intima, media and adventitia)[25], well beyond the resolution power of conventional ultrasound. This enables the site of early vascular changes[11] and progression of disease to be determined, as well as the relation of different lesions with risk factors such as obesity[26], biochemical data, and BP control. UHFUS provides mechanistic insights and permits study of the specific effects of medications, such as statins and phosphate binders.

Early studies have shown that UHFUS can measure differentiated IT and MT in several arterial beds in children[11,26], whereas conventional ultrasound overestimates total combined thickness. It is beneficial to use the highest possible ultrasound frequency for carotid imaging in children. In the present study, carotid imaging was consistently possible at 55 MHz, whereas the more superficial dorsal pedal artery could be visualized with a 70 MHz transducer. It is likely that small vessels in children and early stages of vasculopathy require very high resolution to distinguish between IT and MT and also to detect discrete changes exceeding the yearly increase of 0,004 mm in the IT and 0,006 mm in the IMT of healthy children^26^ in the separate arterial layers with time.

Children in CKD and on dialysis had a significantly higher medial thickness in both carotid and dorsal pedal arteries, compared to transplanted patients, suggesting that the uremic state drives early vascular changes in medial pathology. No intimal changes were seen in CKD or dialysis patients, and this is in keeping with previous clinical and *in vitro* studies of arterial pathology. We and others have shown an increased carotid IMT by conventional US in children on dialysis [5,6,27], but have been unable to determine if this was due to intimal or medial disease. On arterial biopsy samples, we have previously shown changes in the medial layer of the inferior epigastric artery of CKD and dialysis patients, with an increased calcium load and presence of hydroxyapatite crystals[7]. Studies have also confirmed that high serum phosphate levels are consistently associated with increased coronary artery calcification[4–6,19,27] as well as progression of calcification[4]. In the current study, the MT in the common carotid artery reduced significantly from early after transplantation, as uremic related risk factors improved and serum phosphate levels fell to normal. This suggests that there is potential for rapid reversibility of vascular changes after transplantation, in response to a change in the biochemical milieu. UHFUS may provide a non-invasive, radiation-free measure to detect and monitor arterial disease in CKD patients.

In this study, we compared the impact of CKD on arterial wall changes in elastic and muscular arteries in two different vascular beds. We demonstrated increased carotid IMT in CKD and dialysis patients that was exclusively media-driven, but there was a difference in the dorsal pedal artery which showed intimal thickening. The close relationship between uremic risk factors, blood pressure, and carotid MT suggests an increased susceptibility of elastic artery medial changes to the distinct hemodynamic and uremic risk factor profile commonly found in renal failure. Along with increased local inflammation, calcification and thickening of the media in the aorta is already present in early CKD[28]. This is consistent with previous studies in which diffuse arterial calcification and stiffness has been reported in the elastic arteries of adults[29] and children with CKD[6,19]. Compared to elastic arteries, muscular arteries are more dependent on endothelial function for the control of vascular tone[30]. However, this is blunted in lower limb arterial beds due to the added stress of hydrostatic pressure[31], and is compounded by hypertension. The significant elevations in ABPM observed in all kidney patient groups, may have contributed to intimal thickening in the dorsal pedal artery in the current study. As the penetration depth is limited with higher frequency ultrasound, imaging of the more superficial dorsal pedal artery was possible with a higher frequency transducer compared to carotid imaging (70MHz vs 55MHz). This may have allowed more precise measurements of the intima and facilitated demonstration of differences between the study groups. Although the dorsal pedal artery is easier to measure, particularly in obese individuals, in the current study we found stronger correlations between carotid artery measures and risk factors. Further studies to monitor serial changes in the carotid and dorsal pedal arteries in adults and children with CKD are warranted, particularly those on dialysis.

There are of course limitations of this study, with the main being small patient numbers. However, using this very high-resolution ultrasound technique, we were able to show significant changes in the arterial wall at different stages of kidney disease. Although we have described a ‘real world’ situation of children with different stages of CKD who were managed with single center protocols for their renal failure, the effect of different treatment regimens cannot be deduced from our data. Patients did not have significant dyslipidemia, and none were on statins, so the effect of these on vascular measures would need to be assessed in other cohorts of CKD patients who have significant obesity and abnormal lipid profiles. Our study focused on children who have early vascular disease that is more amenable to treatment. Studies in adult CKD, dialysis and transplant patients are required to demonstrate associations between vascular changes and hard endpoints such as mortality and cardiovascular events. In this single center study, we were unable to undertake longitudinal follow-up in dialysis patients as most children had received a renal transplant within the 12-month follow-up period. This will be examined in a future multicenter study.

In conclusion, UHFUS allows distinct imaging of the vascular intima and media, permitting the impact of multiple risk factors on the arterial wall to be assessed at early stages of vascular disease in children with CKD. It has shown that the media bears the brunt of the uremic milieu and elevated blood pressure in children with CKD, particularly in those on dialysis. In contrast, within one year of renal transplantation, changes are detectable in the intimal layer alongside increases in traditional risk factors such as BMI and hypertension. UHFUS imaging therefore offers a highly sensitive tool that can provide important pathophysiological information, and may prove to be useful in tailoring treatments and monitoring response to therapy in clinical practice and interventional trials.

## Supporting information

S1 Table Clinical and biochemical characteristics of the study population at baseline. (PDF)

S2 Table Vascular measures at baseline and 1 year follow-up in CKD and transplanted patients. (PDF)
