# Supplementary material for: Intimal and medial arterial changes defined by ultra-high-frequency ultrasound: Response to changing risk factors in children with chronic kidney disease
Source: PLoS One. 2018 Jun 14;13(6):e0198547. doi: 10.1371/journal.pone.0198547 (PMC6002120; doi:10.1371/journal.pone.0198547)
Supplement: S1 Table — (PDF) [file pone.0198547.s001.pdf]

Supplemental Table 1 Clinical and biochemical characteristics of the study population at baseline

Characteristics	Controls (n = 12)	CKD 4 -5 (n = 19)	Dialysis (n = 20)	Transplant (n = 15)	p
**Clinical features – p values described between controls and patient groups**					
Age (years)	14.0 ± 3.0	13.5 ± 2.7	13.6 ± 4.0	15.5 ± 2.7	0.62
Gender (males; %)	4 (30)	14 (74)	8 (40)	10 (66)	0.09
Ethnic group (Caucasian/ Asian / black / others)	9 / 3 / 0 / 0	9 / 6 / 1 / 3	9 / 8 / 0 / 3	10 / 2 / 0 / 3	0.12
Underlying diagnosis CAKUT / glomerulopathy / other	-	13 / 3 / 3	11 / 4 / 5	9 / 2 / 3	-
Time on dialysis (years)	-	-	2.39 ± 2.4	-	-
Dialysis modality at time of study	-	-	HD 15 / PD 5	-	-
Time with functioning transplant (yr)	-	-	-	3.67 ± 2.24	-
Body Mass Index (BMI) (kg/m^2)<br>BMI - SDS	19.4 ± 2.6<br>-0.04 ± 0.7	21.2 ± 3.9<br>-0.26 ± 0.8	19.1 ± 3.4<br>-0.89 ± 1.2	26.5 ± 8.7<br>2.3 ± 1.8	0.07<br>0.03
Systolic BP (mmHg)<br>SDS	101 ± 6.6<br>-0.06 ± 0.5	110 ± 7.4<br>0.85 ± 1.1	117 ± 10.7<br>2.38 ± 1.0	122 ± 31<br>2.48 ± 2.3	0.02<br>0.02
Diastolic BP (mmHg)<br>SDS	63 ± 5.6<br>-0.11 ± 0.43	66 ± 9.5<br>0.62 ± 1.0	73 ± 10<br>1.64 ± 0.7	66.8 ± 9<br>1.53 ± 1.1	0.08<br>0.02
24-hour mean arterial pressure (mmHg)<br>SDS	-	86 ± 9.7<br>0.71 ± 1.0	92.9 ± 13.4<br>1.83 ± 1.2	102 ± 15.7<br>2.22 ± 1.3	0.01<br>0.01
Number of children on anti-hypertensive medications	0	4	3	9	0.001
Number of anti-hypertensive agents (median, range)	0	0<br>(0 – 2)	1<br>(0 – 2)	2<br>(0 – 2)	-
**Biochemical measures – p values described between CKD and dialysis vs transplant group**					
Estimated GFR (ml/min/1.73m^2)	-	9.63 ± 4.9	-	68.8 ± 22.3	-
Calcium (albumin adjusted; mMol/L)	-	2.36 ± 0.03	2.44 ± 0.18	2.32 ± 0.28	0.71
Phosphate (mMol/L)	-	1.43 ± 0.44	1.76 ± 0.97	1.13 ± 0.28	0.02
Parathyroid hormone (pMol/L)	-	4.7<br>(0.2 – 14.1)	19.8<br>(3.7 – 67.5)	3.0<br>(0.8 – 20.7)	0.01
Albumin (g/L)	-	43 ± 3.2	39.8 ± 4.1	41.7 ± 3.8	0.84
Fibroblast growth factor 23 (RU/ml)	-	237<br>(121 – 476)	1268<br>(566 – 1392)	53<br>(29 – 119)	<0.001
soluble-klotho (pg/mL)	-	1088<br>(462 – 1638)	921<br>(322 – 1341)	1729<br>(1131 –2011)	0.04
25-hydroxyvitamin D (nMol/L)	-	46 ± 27.4	58 ± 31.8	31 ± 50.4	0.08
High sensitivity CRP (mg/L)	-	1.23 ± 0.24	3.11 ± 4.61	1.26 ± 0.20	0.06
Cholesterol<br>Total (mg/dl)<br>HDL (mg/dl)<br>LDL (mg/dl)<br>Triglycerides (mg/dl)	-	 190 ± 36<br>41 ± 5.1<br>153 ± 27<br>123 ± 24	 174 ± 41<br>40 ± 8.7<br>156 ± 37<br>117 ± 28	 220 ± 52<br>46 ± 6.7<br>144 ± 20<br>141 ± 39	 0.11<br>0.72<br>0.65<br>0.26

All data are described as mean ± standard deviation or median (range) where appropriate
SDS – standard deviation score
CAKUT – congenital anomalies of the kidney and urinary tract
HD – hemodialysis; PD – peritoneal dialysis
p values are for comparison across groups as indicated; obtained from ANOVA or Kruskal-Wallis
