# Supplementary material for: Intimal and medial arterial changes defined by ultra-high-frequency ultrasound: Response to changing risk factors in children with chronic kidney disease
Source: PLoS One. 2018 Jun 14;13(6):e0198547. doi: 10.1371/journal.pone.0198547 (PMC6002120; doi:10.1371/journal.pone.0198547)
Supplement: S2 Table — (PDF) [file pone.0198547.s002.pdf]

Supplemental Table 2 Vascular measures at baseline and 1 year follow-up in CKD and transplanted patients

Vascular measures	CKD (n = 16)			Transplant (n = 15)		
	Baseline	1 yr follow-up	p-value	Baseline	1 yr follow-up	p-value
Conventional cIMT (mm)	0.400 ± 0.02	0.44 ± 0.04	0.13	0.45 ± 0.028	0.45 ± 0.04	0.73
cIMT SDS	1.18 ± 0.84	1.37 ± 0.61	0.29	1.88 ± 0.60	1.84 ± 0.71	0.11
Carotid IMT – 55 MHz (mm)	0.388 ± 0.13	0.36 ± 0.06	0.19	0.363 ± 0.037	0.357 ± 0.06	0.49
Carotid IT – 55 MHz (mm)	0.083 ± 0.015	0.075 ± 0.003	0.46	0.078 ± 0.010	0.085 ± 0.011	0.06
Carotid MT – 55 MHz (mm)	0.298 ± 0.02	0.271 ± 0.058	0.38	0.293 ± 0.018	0.245 ± 0.025	0.01
Dorsal pedal IMT– 70 MHz (mm)	0.137 ± 0.018	0.135 ± 0.024	0.58	0.134 ± 0.030	0.151 ± 0.043	0.33
Dorsal pedal IT – 70 MHz (mm)	0.061 ± 0.009	0.065 ± 0.007	0.32	0.058 ± 0.010	0.068 ± 0.011	0.04
Dorsal pedal MT – 70 MHz (mm)	0.086 ± 0.018	0.077 ± 0.022	0.56	0.076 ± 0.029	0.086 ± 0.042	0.45

IMT – intima – media thickness; IT – intimal thickness; MT – medial thickness; cIMT – carotid IMT; SDS – standard deviation score;
p-value between baseline and follow–up by paired t-test
